# Isolation and Analysis of B-cell Progenitors from Bone Marrow by Flow Cytometry

**DOI:** 10.21769/BioProtoc.4835

**Published:** 2023-10-05

**Authors:** Hongchang Zhao, Roger Sciammas, Jacqueline H. Barlow

**Affiliations:** 1 Department of Emergency Surgery, The First Affiliated Hospital of Bengbu Medical College, Bengbu, China; 2 Department of Anatomy, Physiology, and Cell Biology, University of California Davis, Davis, CA, USA; 3 Department of Microbiology and Molecular Genetics, University of California Davis, Davis, CA, USA

**Keywords:** B-cell development, Lymphocyte staining, Bone marrow isolation, Adaptive immunity, Flow cytometry

## Abstract

B cells play a critical role in host defense, producing antibodies in response to microbial infection. An inability to produce an effective antibody response leaves affected individuals prone to serious infection; therefore, proper B-cell development is essential to human health. B-cell development begins in the bone marrow and progresses through various stages until maturation occurs in the spleen. This process involves several sequential, complex events, starting with pre- and pro-B cells, which rearrange the heavy and light chain genes responsible for producing clonally diverse immunoglobulin (Ig) molecules. These cells then differentiate into immature B cells, followed by mature B cells. The bone marrow is a complex ecological niche of supporting stromal cells, extracellular matrix components, macrophages, and hematopoietic precursor cells influencing B-cell development, maturation, and differentiation. Once fully mature, B cells circulate in peripheral lymphoid organs and can respond to antigenic stimuli. As specific cell surface markers are expressed during each stage of B-cell development, researchers use flow cytometry as a powerful tool to evaluate developmental progression. In this protocol, we provide a step-by-step method for bone marrow isolation, cell staining, and data analysis. This tool will help researchers gain a deeper understanding of the progression of B-cell development and provide a pertinent flow gating strategy.

## Background

**Figure 1.**
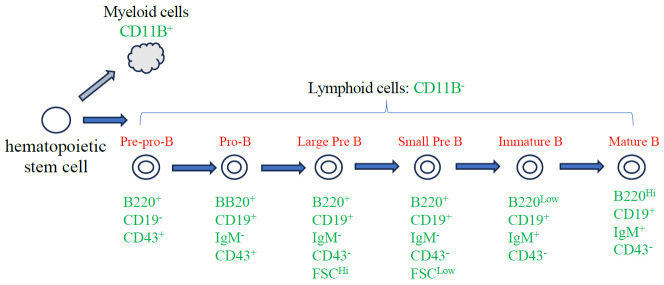
Diagram of B-cell development. Major stages of adult B-cell development in the bone marrow focusing on the cell surface markers relevant to this protocol.

Hematopoietic stem cells (HSCs) produce all cellular components of the blood through a series of unequal cellular divisions in the bone marrow. HSCs give rise to common myeloid progenitor and common lymphoid progenitor (CLP) cells that give rise to both T and B lymphocyte lineages. B cell–specific development starts when CLPs give rise to progenitor B cells. Progenitor B cells first differentiate into pro-B cells, which then undergo a series of cell division and differentiation events to create immature B cells (reviewed in [1,2]). Distinct surface receptors are expressed at each stage of B-cell development, allowing investigators to *mark* successive stages of development: B220 and CD43 are expressed on pre-pro-B cells, the earliest stage of B-cell commitment; CD19 expression becomes visible on early pro-B cells and is retained throughout development; finally, CD43 expression is lost on pre-B cells [3–7] (Figure 1).

These stages are also defined by successive VDJ and VJ recombination events of the immunoglobulin heavy (IgH) and immunoglobulin light (IgL) chains gene loci, respectively, leading to IgM expression [8–10]. Upon successful assembly of the B-cell receptor (BCR), B cells migrate to secondary lymphoid organs and await their chance to encounter antigen. Antigen stimulation of the BCR results in a receptor signaling cascade that instructs cell activation pathways including changes in gene expression, G0–G1 progression, and bioenergetic metabolism in preparation for clonal expansion. Critically, the BCR simultaneously delivers antigenic cargo to endocytic processing compartments where proteolytic fragments are loaded onto major histocompatibility antigen class II (MHCII) and recycled to the cell surface as peptide:MHC complexes. T-cell recognition of such peptide:MHCII results in release of cell surface CD40L and secretion of the IL21 on activated T cells. Stimulation of CD40 and IL21R signaling on B cells by activated T cells licenses clonal expansion. Clonal expansion and coordinated changes in gene expression promote B-cell differentiation and B-cell fate choices into plasma cells, germinal center B cells, or memory B cells [11]. The distinct activity of these cell fates comprises the antibody response. Importantly, some clones will also undergo DNA class switch recombination, whereby the exons of the constant region segment (not the antigen combining site VDJ/VJ exons) are exchanged for a different one, e.g., μ to γ, ε, or α while retaining original antigen specificity. Antibodies of different isotypes exhibit different functionality, including fixation of complement, opsonization, or antibody-dependent cellular cytotoxicity to elaborate the general immune response. Because antibody responses are fundamental to host protection from pathogenic infections, the field has intensively studied the generation of B cells and their subsequent activation and differentiation. In this protocol, we focus on B-cell development and provide a time-tested flow cytometry–based assay for evaluating successive stages of B-cell development in the bone marrow [12–15]. Thus, this protocol will be useful for the evaluation of B-cell development in different mouse models as well as for the interrogation of discrete events in each stage [16]. This protocol describes the details of bone marrow isolation, cell surface antibody labeling to differentiate distinct developmental stages, and the flow cytometry gating strategy to quantify each population.

## Materials and reagents

1. Scissors (VWR, catalog number: 82027-578)
2. Tweezers (VWR, catalog number: 82027-398)
3. Dissection pins (VWR, catalog number: 76549-022)
4. 70% ethanol
5. Kimwipe task wipers (Kimtech brand, Amazon)
6. Pipette tips (non-filter)
7. 18 G syringe needle (Fisher Scientific, catalog number: 14-826)
8. 0.22 μm syringe filter (Fisher Scientific, catalog number: SLHP033RS)
9. Biohazard bag(s)
10. CO_2_ tank
11. 0.5 mL microcentrifuge tubes (VWR, catalog number: 0011-830)
12. 1.5 mL microcentrifuge tubes (Genesee Scientific, catalog number: 24-281)
13. 70 μm mesh net cut into ~1.5 cm squares to cover tube opening (Component Supply Company Inc, U-CMN-70); 70 μm filters can be used instead (Fisher Scientific, catalog number: 07201431)
14. Bovine serum albumin (BSA) (Sigma, catalog number: BP1600)
15. NaCl (Sigma, catalog number: BP358)
16. KCl (Sigma, catalog number: P9541)
17. Na_2_HPO_4_ (Sigma, catalog number: S374500)
18. KH_2_PO_4_ (Sigma, catalog number: P9791)
19. Sodium azide (Sigma, catalog number: BP922I)
20. NH_4_Cl (Sigma, catalog number: A661)
21. KHCO_3_ (Sigma, catalog number: P235)
22. EDTA-Na_2_ (Fisher BioReagents, catalog number: BP120-1)
23. Counting beads (Spherotech, catalog number: ACBP-50-10)
24. Mice (C57BL/6, C57BL/6 × 129/Sv)
25. Anti-mouse CD16/CD32 (Fc shield) antibody (2.4G2) [Tonbo (now Cytek), catalog number: 70-0161-U100]
26. Rat anti-mouse monoclonal, APC CD19 (BD Pharmingen, catalog number: 561738)
27. Rat anti-mouse monoclonal, PE-Cy7-CD43 (BD Pharmingen, catalog number: 562866)
28. Rat anti-mouse monoclonal, PE-IgM (BD Pharmingen, catalog number: 562033)
29. Rat anti-mouse monoclonal, BV421-CD11b (BD Pharmingen, catalog number: 562605)
30. Rat anti-mouse monoclonal, PerCP-CD45R/B220 (BD Pharmingen, catalog number: 553093)
31. Wash buffer (see Recipes)
32. 1× PBS buffer (see Recipes)
33. ACK (ammonium-chloride-potassium) lysis buffer (see Recipes)

**Recipes**

1. **Wash buffer**
  - Sterile PBS (1×)
  - 1% BSA
  - 0.05% sodium azide
  Dissolve 1 g of BSA powder and 0.05 g of sodium azide in 100 mL of PBS (1×) to make the wash solution. Filter the solution through a 0.22 μm syringe filter and store it at 4 °C for up to one week.
2. **1× PBS buffer**
  - NaCl 137 mM
  - KCl 2.7 mM
  - Na_2_HPO_4_ 10 mM
  - KH_2_PO_4_ 1.8 mM
    To prepare 1 L of 1× PBS, dissolve the reagents listed above in 800 mL of H_2_O. Adjust the pH to 7.4 with HCl, then add H_2_O to 1 L. Sterilize by autoclaving for 20 min and store at room temperature (RT) for up to one year.
3. **ACK buffer**
  - 8.29 g of NH_4_Cl (0.15 M final concentration)
  - 1 g of KHCO_3_ (1 mM final concentration)
  - 37.2 mg of Na_2_EDTA (0.1 mM final concentration)
  Add 800 mL of H_2_O and adjust the pH to 7.2–7.4 with 1 N HCl (sterilize by filtration using a 0.2 μm filter)
  Store at RT for up to one year.

## Equipment

1. Vortex (VWR, model: 88880017)
2. Centrifuge (Eppendorf, model: 5424R)
3. Pipettes (Eppendorf)
4. Tissue culture light microscope equipped with brightfield and 20× objective (Olympus CK-40)
5. Flow cytometer (LSR II, BD)

## Software

1. FlowJo software (FlowJo, LLC.)
2. Prism 6 software (GraphPad Software, Inc.)

## Procedure

- **Euthanize the mice and isolate the femur**
  1. Euthanize the mice with CO_2_ under standard protocol [17] or to the specifications of the users’ mouse facility.
  2. Pin the mouse onto a foam board with needles in a supine position (Figure 2).
    **Figure 2.**
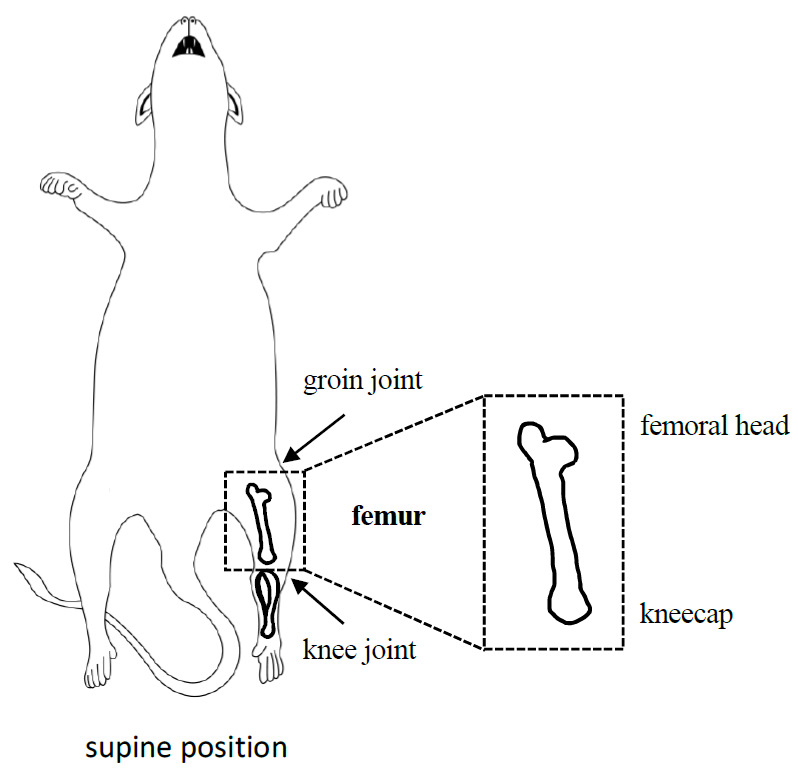
Mouse dissection diagram focusing on the location and isolation of intact femur
  3. Spray the mouse with 70% ethanol.
  4. Make an incision on the hind leg and extend it from foot to groin.
  5. Pull the skin to the back and cut it to expose the muscle.
  6. Carefully remove the muscle with scissors from knee joint to groin joint.
  7. Cut the knee joint and groin joint with scissors.
  8. Isolate the femur containing the kneecap and femoral head.
*Note: Keep the integrity of the kneecap and femoral head (rounded with less opaque cartilage) to make sure the bone marrow is not released from femur.*
  9. Remove extra muscle tissue with Kimwipes so there is no lint.
  10. Repeat with second hind leg to isolate two femurs in total.
  11. Discard mouse carcass according to UC Davis Institutional Animal Care and Use Committee (IACUC protocol #21828).
- **Bone marrow isolation**
  1. Puncture the bottom of a 0.5 mL microcentrifuge tube by hand from the inside of the tube with an 18 G needle and place it into a 1.5 mL microcentrifuge tube.
*Note: Take care and wear thick protective gloves.*
  2. Cut both ends of the femur with scissors and place them in the 0.5 mL tube.
  3. Centrifuge the tube at 2,000× *g* for 10 s at RT.
  4. Discard the 0.5 mL centrifuge tube.
  5. Resuspend the bone marrow cells with ACK lysis buffer for 2 min at RT.
  6. Centrifuge the cells at 500× *g* for 5 min at RT.
  7. Throw away the supernatant, resuspend the cells with 1 mL of wash buffer, and filter the cells through the mesh net.
  8. Count the cells. **Potential stop point:** Isolated bone marrow cells can be stored at 4 °C for a few hours.
*Note: We recommend preparing 4 million cells per staining. Less than 1 million cells can be used per staining; however, this may cause quantification problems after flow cytometry.*
- **B-cell staining**
  1. Prepare antibody staining solutions: dilute CD11B, B220, IgM, and CD19 antibodies 1:99 in wash buffer; note that the CD43 antibody is not diluted. The fluorochrome combination used below was optimized for use on cytometers (BD Fortessa and Sony SH800S) with at least three lasers: blue (488 nm), red (640 nm), and violet (405 nm). Fluorochrome combinations should be chosen based on the instrument used and its laser availability; consultation with flow core personnel to minimize emission overlap is strongly recommended.
  2. Pipette the cell suspension obtained after isolation into individual sample tubes; we recommend 4 × 10^6^ cells for each experimental sample. For the full five antibody staining, prepare 11 additional control samples following the table below [no stain, single antibody staining, multi-antibody staining, and fluorescence minus one (FMO) staining controls].
  3. Adjust the volume with wash buffer to 100 μL/sample.
  4. Add 10 μL (1:99 diluted) of anti-FC blocking antibody to each staining tube.
  5. Add 10 μL of prepared antibody solution for CD11B, B220, IgM, and CD19 to all experimental samples and the relevant control tubes. Add 1 μL of undiluted CD43 antibody to all experimental samples and the relevant control tubes.
  6. Incubate in the dark for at least 1 h.
  7. Centrifuge the sample at 1,000× *g* for 2 min at RT.
  8. Wash twice with 1 mL of wash buffer and resuspend with 490 μL of wash buffer.
  9. Add 10 μL of counting beads (invert several times before pipetting the beads) before flow cytometry.
    **Table 1.** Representative sample panel for five color staining including all control samples necessary for compensation. No stain (no fluorescent antibody, blocking antibody only), S (single antibody staining), FMO (fluorescence minus one staining). *Volume of antibody used relative to the original concentration from the manufacturer.

		Staining combination											
**Antibody**	Volume*	**No stain**	**S1**	**S2**	**S3**	**S4**	**S5**	**Testing sample**	**FMO1**	**FMO2**	**FMO3**	**FMO4**	**FMO5**
BV421-CD11B	0.1 μL	-	+	-	-	-	-	+	-	+	+	+	+
FITC-B220	0.1 μL	-	-	+	-	-	-	+	+	-	+	+	+
PE-IgM	0.1 μL	-	-	-	+	-	-	+	+	+	-	+	+
APC-Cy7-CD43	1 μL	-	-	-	-	+	-	+	+	+	+	-	+
APC-CD19	0.1 μL	-	-	-	-	-	+	+	+	+	+	+	-
- **Flow cytometry operation**
  1. The *no stain* sample is used to set laser voltages, the single-color staining samples are used to compensate fluorochrome emission overlap, and the FMO samples are used to provide empirical evidence that the instrument was well compensated. Fluorochrome emission is often broad and the broad emission spectra overlap potentially creating false positive signals. Instrument compensation, which superficially is a mathematical-based subtraction of fluorescence signal in a distinct channel, is essential for proper interpretation of the results. In the preliminary run, perform flow cytometry with an experienced user or flow core manager to help with compensation so samples are not over- or under-compensated.
  2. When running each single-color-stained sample, check that other desired channels display minimal emission, i.e., if a sample is stained with an APC-Cy7-conjugated antibody alone, there should not be any signal in the FITC channel. If there is, then instrument compensation is in order and entails increasing compensation such that detection in the FITC channel is eliminated; in this case, the mean fluorescence intensity on the FITC axis of the APC-Cy7-stained population is equivalent to the non-APC-Cy7-stained population. Repeat with each single-color-stained sample against all other desired channels (Figure 3).
    **Figure 3.**
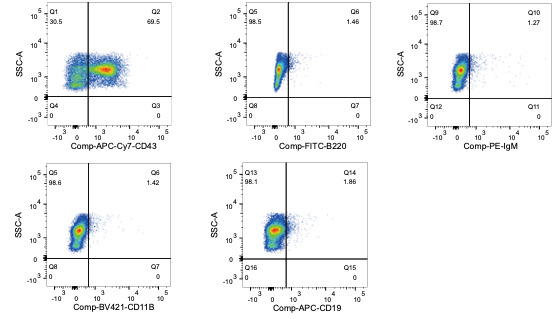
Representative flow cytometry graphs post-compensation using the APC-Cy7-CD43 staining. Isolated bone marrow B cells were stained with APC-Cy7-CD43 antibody following the protocol, to determine if it is detected by other channels being used (FITC, PE, BV-421, and PE); every channel is checked separately. This confirms the APC-Cy7-CD43 positive (69.5%) population is being identified in the appropriate channel, while compensation in other single channels eliminates/strongly reduces the bleed-through signal from overlap in spectral emission.
  3. Run and record all samples, including all controls, after compensation adjustment.
*Note: Current analysis software can use digital compensation to adjust compensation post-sample acquisition; however, given that laser voltage is intimately correlated with emission, i.e., higher voltages result in greater emission and thus more overlap, performing pre-acquisition compensation is an important practice for result quality. Recording all controls maintains a record of the staining quality and allows for digital re-adjustment of compensation later.*
- **Data analysis using FlowJo software**
  Gate the beads and the cells according to Figure 4. If beads are included, one can calculate the absolute number of cells of a given population that is designated by a given gate. This is helpful in understanding whether frequencies of developmental progenitors are unchanged, but progeny output is diminished. Specifically, the absolute number of B cells at different stages of development is calculated by: (number of events for the test samples/number of events for the counting beads) × (number of beads used/volume of test sample initially used).
  **Figure 4.**
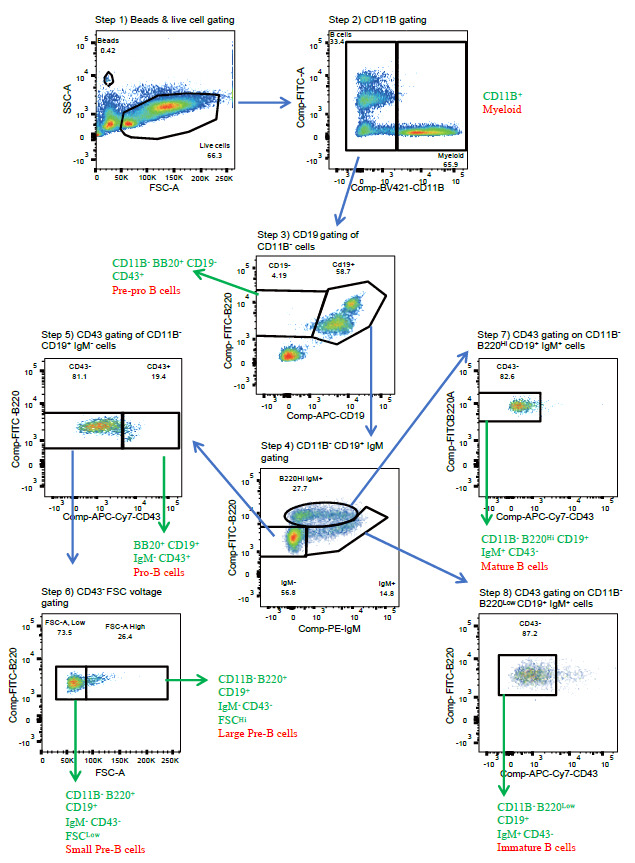
Representative flow cytometry plots for each stage of the gating pipeline for data analysis. Sample is from a wildtype mouse.
  Gating pipeline (see Figure 4):
  Step 1. Gate on live large cells using the forward and side scatter channels (FSC and SSC, respectively).
  - Avoid fragmented and dead cells.
  *Optional: if quantitation is desired, gate beads to calculate absolute cell numbers.
  Step 2. Distinction between myeloid vs. lymphoid cells is defined here as CD11B^+^/CD11B-.
  - CD11B^+^ cells: myeloid—should be low for B220 signal.
  - Lymphoid cells: CD11B-negative—these are further gated for B-cell developmental stages.
  Step 3. Distinction between pre-pro B cells and later stages is defined here by the density of B220 and CD19 expression within the CD11B- population.
  - B220^+^ CD19- are pre-pro B cells.
  - B220^+^ CD19^+^ comprise a mixture of more differentiated B cells (steps 4 and 5 below).
  Step 4. Pro-, small pre-, large pre-, and mature B cells are distinguished based on IgM expression within the B220^+^ CD19^+^ population.
  - IgM- cells comprise pro-, small pre-, and large pre-B cells based on CD43 and FSC parameters (steps 5 and 6).
  - IgM^+^ comprise immature and recirculating mature B cells based on their B220 density (steps 7 and 8, respectively).
  Step 5. Pro-, small pre-, and large pre-B cells are distinguished between levels of CD43 expression.
  - Pro-B cells display the greatest CD43 expression.
  - Small pre- and large pre-B cells comprise the CD43- population and can be distinguished by size (FSC); see step 6.
  Step 6. Small and large pre-B cells are distinguished based on the FSC spread.
  - Large pre-B cells exhibit the greatest FSC; these cells express a complex of a rearranged heavy chain paired with surrogate light chain, also known as the pre-BCR.
  - Small pre-B cells display the lowest FSC and follow large pre-B cells and are in the process of rearranging the VJ segments of the light chain.
  Step 7. Mature recirculating B cells have downregulated CD43 expression among cells defined by the gate in step 4b; B220^Hi^ CD19^+^ IgM^+^ cells.
  Step 8. Immature B cells that express a functional surface BCR and are migrating to secondary lymphoid organs have downregulated CD43 expression among cells defined by the gate in step 4b; B220^Low^ CD19^+^ IgM^+^ cells.

## Validation of protocol

The protocol presented here used data acquired from the bone marrow of a wildtype mouse (Figures 3 and 4) and was used in [16].
